# Supplementary material for: Polymorphism and evolutionary origins of accessory chromosomes in the basidiomycete Tremella fuciformis
Source: Nat Commun. 2026 Feb 27;17:3275. doi: 10.1038/s41467-026-70078-y (PMC13066641; doi:10.1038/s41467-026-70078-y)
Supplement: Supplementary file 1 — Supplementary Information [file 41467_2026_70078_MOESM1_ESM.pdf]

Supplementary information for

Polymorphism and evolutionary origins of accessory chromosomes in the basidiomycete *Tremella fuciformis*

Jinxiang Zhang[1,#], Qianwen Tong[1,#], Fengjiao Lin[1,#,] Xingcai An[2], Haichen Huang[1], Hualian Chen[1], Jingjing Ye[1], Huaizhen Xu[1], Xiaomeng Lv[1], Zhiwen Lv[3], Fangting Zeng[1], Tuo Zhang[1], Xiaoping Wu[1], Baogui Xie[1*], Ray Ming[4,5*], Youjin Deng[1*]

The supplementary information includes:

Supplementary Table 1-7

Supplementary Figure 1-14

Supplementary Table 1. Source information of *T. fuciformis* strains.

Strain ID	Source Location	Type	Morphotype	Nuclear Status	Associated *A. stygium*
TF2206	Tongjiang County, Bazhong, Sichuan, China	Wild	M/Y	Heterokaryon	+
TF4-1	Tongjiang, Bazhong, Sichuan, China	Wild	M/Y	Heterokaryon	+
QC	Tongjiang, Bazhong, Sichuan, China	Wild	M/Y	Heterokaryon	+
TF103	Tongjiang, Bazhong, Sichuan, China	Wild	M/Y	Heterokaryon	+
TF104*	Tongjiang, Bazhong, Sichuan, China	Wild	M/Y	Haploid	+
NDB	Mangdang Mountain, Nanping, Fujian, China	Wild	M/Y	Heterokaryon	+
QS	Qishan National Forest Park, Fuzhou, Fujian, China	Wild	M/Y	Heterokaryon	+
Wuyi2015	Wuyishan Biosphere Reserve, Nanping, Fujian, China	Wild	Y	Haploid	-
Wuyi2014	Wuyishan Biosphere Reserve, Nanping, Fujian, China	Wild	Y	Haploid	-
Tr01	Gutian County, Ningde, Fujian, China	Cultivated	M/Y	Heterokaryon	+
Tr21	Gutian County, Ningde, Fujian, China	Cultivated	M/Y	Heterokaryon	+
GD	Shaoguan, Guangdong, China	Wild	M/Y	Heterokaryon	+
B-1	Tengchong, Yunnan, China	Wild	M/Y	Heterokaryon	+
C-1	Tengchong, Yunnan, China	Wild	M/Y	Heterokaryon	+
LYS2020	Liangyeshan Mountain, Wuping County, Longyan, Fujian, China	Wild	Y	Haploid	-
T0053	Wuping Longyan, Fujian, China	Wild	Y	Haploid	-

Note: M/Y indicates that the strain exhibits both mycelial (M) and yeast-like/ (Y) morphological states; + indicates presence of an associated *A. stygium* strain; - indicates absence.

*: Samples used for genomic analysis were derived from a single purified yeast colony of heterokaryotic mycelia, confirmed to be haploid (presumably transitioning from the heterokaryotic to haploid state). In contrast, samples employed for association assay with *A. stygium* were heterokaryotic mycelia.

Supplementary Table 2. *T. fuciformis* genome assembly statistics using HiFi.

Strain ID	HiFi reads number	Total data (Gb)	Contig N50 (Kb)	Coverage (×)
TF2206	540,905	10.58	19.71	441
TF4-1	361,242	6.76	18.78	273
QC	247,810	5.42	22.15	222
TF103	332,323	6.34	19.58	259
TF104	321,258	6.89	21.97	282
NDB	329,762	7.27	22.06	232
QS	393,337	8.87	22.49	265
Wuyi2015	180,615	3.37	18.44	103
Wuyi2014	406,556	4.07	12.25	131
Tr01	211,688	3.34	21.17	120
Tr21	575,272	9.52	16.96	142
GD	389,229	9.21	23.57	321
B-1	365,840	7.87	21.6	286
C-1	280,680	5.97	21.31	221
LYS2020	286,678	4.47	15.67	159
T0053	224,771	3.96	17.27	139

Supplementary Table 3. Information of Starships identified in 27 genomes of *T. fuciformis*.

Starship ID	Haplotype ID	Length (bp)	YR count	Captain ID	Source species	Source strain ID	Source chromosome	Chromosome type	Start	End	Strand
SS01	TF4-1A	605,797	1	Tf_tyr11	*Tremella fuciformis* Berk.	TF4-1	Chr05A	CC	896,488	1,502,284	-
SS02	C-1A	41,942	1	Tf_007298	*Tremella fuciformis* Berk.	C-1	Chr06A	CC	1639,952	1,681,893	-
SS03	C-1B	41,942	1	Tf_008157	*Tremella fuciformis* Berk.	C-1	Chr06B	CC	1,689,388	1,731,329	-
SS04	GDA	174,777	1	Tf_tyr25	*Tremella fuciformis* Berk.	GD	Chr09A	AC	356,504	531,280	-
SS05	GDB	177,826	1	Tf_tyr27	*Tremella fuciformis* Berk.	GD	Chr09B	AC	345,354	523,179	-
SS06	C-1B	297,775	1	Tf_010117	*Tremella fuciformis* Berk.	C-1	Chr10B	AC	1,046,099	1,343,873	+
SS07	C-1A	297,822	1	Tf_tyr21	*Tremella fuciformis* Berk.	C-1	Chr10A	AC	1,046,044	1,343,865	+
SS08	LYS2020	83,112	1	Tf_tyr10	*Tremella fuciformis* Berk.	LYS2020	Chr10A	AC	328,924	412,035	+

Note: CC = core chromosome; AC = accessory chromosome.

Supplementary Table 4. Statistical definitions and sample size information for box plots in Fig. 3a-d/Fig.S12A-C.

Figure	Group	Category	Metric	n	Replicate type	Unit of study	Centre	Box bounds	Whiskers
Fig.3a-d/ Fig.S12 A-C	CC	CC01	Length / Gene density /TE proportion/ OrthoANIu/ Length/Number	27	Biological	Individual core chromosome within predefined chromosome groups (CC01-CC08)	Median	25-75 percentile	Min-max
		CC02		27					
		CC03		27					
		CC04		27					
		CC05		29					
		CC06		27					
		CC07		27					
		CC08		27					
	AC	AC01	Length / Gene density / TE proportion /OrthoANIu /Length/Number	6		Individual accessory chromosome within predefined chromosome groups (AC01-AC15)			
		AC02		8					
		AC03		4					
		AC04		8					
		AC05		6					
		AC06		5					
		AC07		8					
		AC08		7					
		AC09		5					
		AC10		7					
		AC11		7					
		AC12		9					
		AC13		10					
		AC14		11					
		AC15		14					

Note: During boxplot construction and statistical analysis, homologous chromosomes were assigned to their respective chromosome groups (CC01-CC08 or AC01-AC15) and treated as the constituent units of each group. Both AC02 and AC07 contain eight chromosomes; among them, seven chromosomes in AC02 exhibit clear one-to-one homologous relationships with their corresponding chromosomes in AC07 at partial sequence levels. Accordingly, the boxplot results primarily reflect overall distributional differences in the analyzed features among chromosome groups, rather than assuming complete statistical independence among individual chromosome data points.The sample size (n) for each category indicates the number of chromosomes included in that group. Definitions of medians, interquartile ranges, whiskers, and outliers are consistent with those described in the corresponding figure legends. All data points represent biological replicates, and no technical replicates were used.

Supplementary Table 5. Statistical definitions and sample size information for box plots in Fig. 5a.

Figure	Category	Metric	n	Replicate type	Unit of study	Centre	Box bounds	Whiskers
Fig.5a	1-1-A	Depth	2110	Biological	Fixed-length (1 Kb), non-overlapping genomic window along an individual chromosome from a single colony	Mean	25-75 percentile	Min-max
	1-2-YY3		610					
	1-2-O		5490					
	2-1-A		2030					
	2-2-A		1570					
	3-A		2240					
	4-A		1890					
	5-YY3		171					
	5-O		1539					
	6-A		1950					
	7-A		1970					
	8-A		1300					
	9-A		1110					
	10-A		90					
	11-YY1		9					
	11-YY8		9					
	11-O		72					

Supplementary Table 6. Statistical definitions and sample size information for box plots in Fig. 5b.

Figure	Category	Metric	n	Replicate type	Unit of study	Centre	Box bounds	Whiskers
Fig.5b	1-1-A	Depth	1899	Biological	Fixed-length (1 Kb), non-overlapping genomic window along an individual chromosome from a single colony	Mean	25-75 percentile	Min-max
	1-2-A		5490					
	2-1-A		1827					
	2-2-A		1413					
	3-A		2016					
	4-A		1701					
	5-A		1539					
	6-A		1755					
	7-A		1773					
	8-A		1170					
	9-M6/9		222					
	9-O		777					
	10-M2		9					
	10-O		72					
	11-M1		9					
	11-M2		9					
	11-M3/9		18					
	11-M4/8/10		27					
	11-M6/7		18					

Supplementary Table 7. Statistical definitions and sample size information for box plots in Fig. 5c.

Figure	Category	Metric	n	Replicate type	Unit of study	Centre	Box bounds	Whiskers
Fig.5c	1-1-A	Depth	2110	Biological	Fixed-length (1 Kb), non-overlapping genomic window along an individual chromosome from a single colony	Mean	25-75 percentile	Min-max
	1-2-A		6100					
	2-1-A		2030					
	2-2-MY1		157					
	2-2-MY10		157					
	2-2-MY2/7		314					
	2-2-MY4		157					
	2-2-MY5/6		314					
	2-2-O		471					
	3-A		2240					
	4-A		1890					
	5-MY6		171					
	5-O		1539					
	6-A		1950					
	7-A		1970					
	8-MY3		130					
	8-MY6/9		260					
	8-O		910					
	9-MY10/7		222					
	9-MY2/5		222					
	9-O		666					
	10-MY1		9					
	10-MY2/6		18					
	10-MY3		9					
	10-MY7		9					
	10-MY8/9		18					
	10-O		27					
	11-MY2		9					
	11-MY8/9		18					
	11-O		63					

Note: Boxplots for each chromosome (C1-1 to C11) were generated from short-read sequencing data of individual colonies. Reads were aligned to the reference genome, and normalized sequencing depth was calculated using 1 Kb non-overlapping fixed genomic windows. Each data point represents the mean sequencing depth of a single 1 Kb window per chromosome, with sample size (n) corresponding to the number of 1 Kb windows on each chromosome. In boxplots, boxes denote the interquartile range ((25th-75th percentiles), the central line indicates the mean, and whiskers extend to the minimum and maximum values within 1.5×IQR; values outside this range are plotted as outliers. All boxplots are based on biological replicates (independent colonies) with no pooling of

technical replicates. This analysis characterizes chromosome-level copy number variation patterns within individual colonies, and no direct statistical comparisons were performed among colonies.

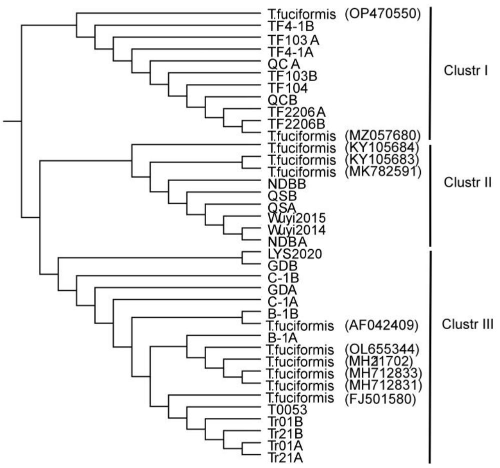

Supplementary Figure 1. Maximum Likelihood phylogenetic tree of 16 *T. fuciformis* strains based on ITS sequences.

Note: Eleven ITS sequences (MZ057680, OP470550, KY105683, MK782591, AF042409, FJ501580, OL655344, MH712833, KY105684, MH211702, MH712831) were retrieved from the online UNITE database (https://unite.ut.ee/schedule_analysis.php) as the most similar homologous sequences to the ITS sequences of 16 *T. fuciformis* strains haploid genomes.

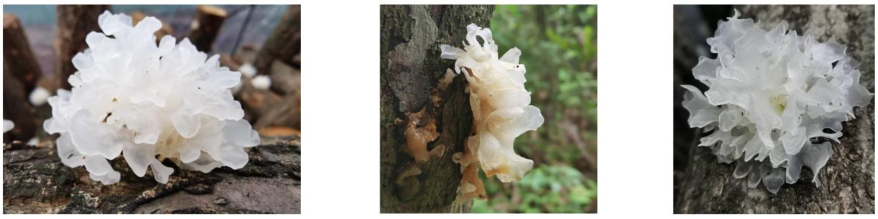

TF2206 QS Tr21

Supplementary Figure 2. Field-collected fruiting bodies of representative strains from three Clusters of *T. fuciformis.*

The images display wild fruiting bodies of typical strains from Cluster I (TF2206), Cluster II (QS), and Cluster III (Tr21), which were selected from the 16 analyzed strains in this study. All specimens were collected from natural habitats in China and photographed under native growth conditions.

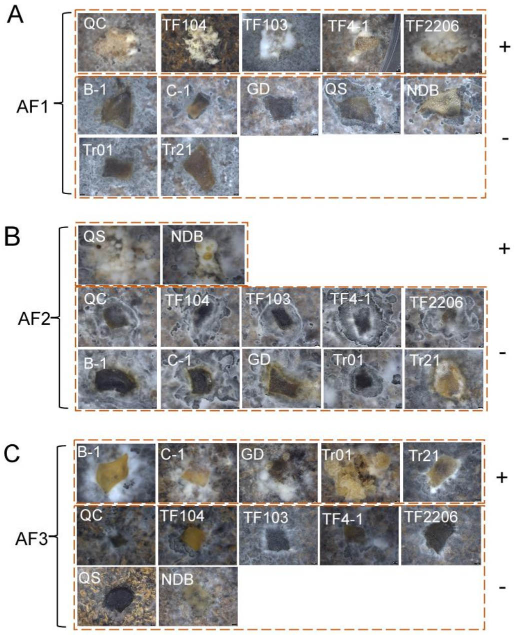

Supplementary Figure 3. Pairing compatibility between *T. fuciformis* strains and their associated fungus (AF) across phylogenetic clusters.

(A-C) Representative *T. fuciformis* strains from Cluster I **(A)**, Cluster II **(B)**, and Cluster III **(C)**, showing successful pairing (indicated by +) with their native AF isolates (e.g., AF1, AF2, AF3), but incompatibility with strains from other clusters.

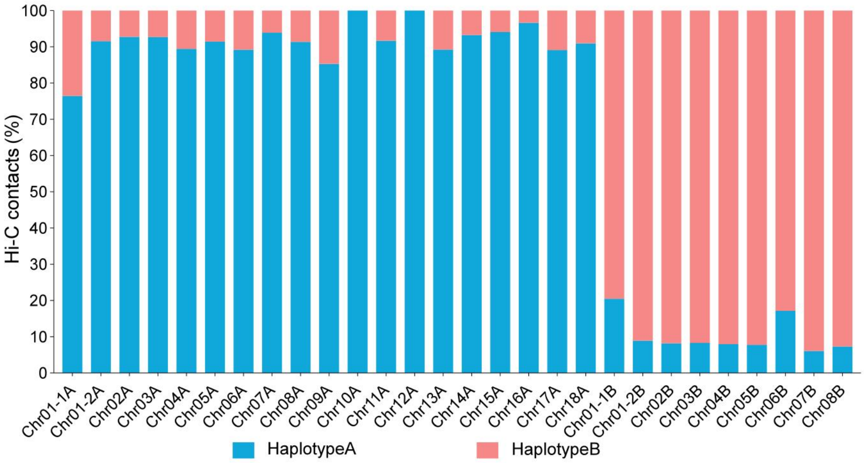

Supplementary Figure 4. NDB Haplotype-resolved Hi-C contact profiles.

Heatmap depicts the distribution of Hi-C contact frequencies (%) between haplotypeA and haplotypeB across 18 chromosomes (Chr01-Chr18). The x-axis indicates chromosome numbers (suffix A/B denotes haplotype assignment), while the y-axis represents normalized Hi-C contact frequency (%). Color gradient reflects haplotype-specific interaction intensity, with haplotypeA (blue) and haplotypeB (red) showing distinct interaction partitioning.

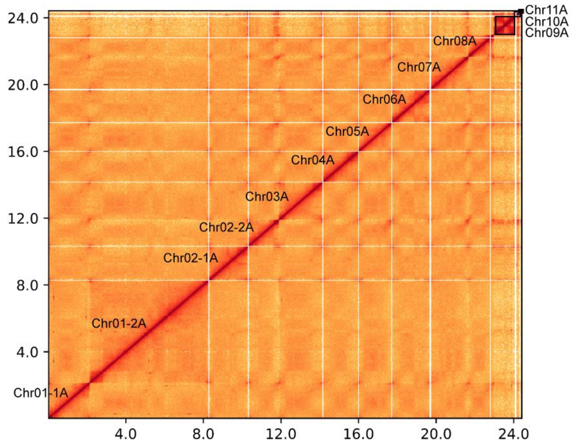

Supplementary Figure 5. Hi-C interaction heatmaps produced by Hi-C Pro validated the assembled genomic architecture of TF2206.

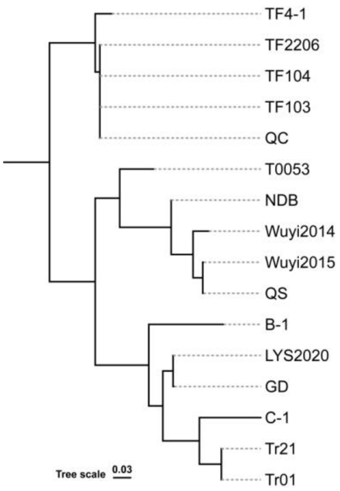

Supplementary Figure 6. Maximum likelihood phylogenetic tree of mitochondrial genomes based on complete genome sequences of 16 strains.

	Tr01		Tr21		B-1		C-1		T0053	LYS2020	GD		Wuyi 2014	Wuyi 2015	QS		NDB		TF103		TF2206		TF104	QC		TF4-1	
Tr01A	A	B	A	B	A	B	A	B	A	A	A	B	A	A	A	B	A	B	A	B	A	B	A	A	B	A	B
Chr01A	+	+	+	+	+	+	+	+	+	+	+	+	+	+	+	+	+	+	+	+	+	+	+	+	+	+	+
Chr02A	+	+	+	+	+	+	+	+	+	+	+	+	+	+	+	+	+	+	+	+	+	+	+	+	+	+	+
Chr03A	+	+	+	+	+	+	+	+	+	+	+	+	+	+	+	+	+	+	+	+	+	+	+	+	+	+	+
Chr04A	+	+	+	+	+	+	+	+	+	+	+	+	+	+	+	+	+	+	+	+	+	+	+	+	+	+	+
Chr05A	+	+	+	+	+	+	+	+	+	+	+	+	+	+	+	+	+	+	+	+	+	+	+	+	+	+	+
Chr06A	+	+	+	+	+	+	+	+	+	+	+	+	+	+	+	+	+	+	+	+	+	+	+	+	+	+	+
Chr07A	+	+	+	+	+	+	+	+	+	+	+	+	+	+	+	+	+	+	+	+	+	+	+	+	+	+	+
Chr08A	+	+	+	+	+	+	+	+	+	+	+	+	+	+	+	+	+	+	+	+	+	+	+	+	+	+	+
Chr09A	+	+	+	+	+	+	+	+	+	+	-	-	-	-	-	-	-	-	-	-	-	-	-	-	-	-	-
Chr10A	+	+	+	+	+	+	+	+	-	+	+	+	-	-	-	-	-	-	-	-	-	-	-	-	-	-	-
Chr11A	+	+	+	+	-	-	-	-	+	+	+	+	+	+	+	+	+	+	-	-	-	-	-	-	-	-	-

Supplementary Figure 7. Synteny analysis of chromosomes between the Tr01 hapA genome and 26 haploid genomes.

The + symbol on a blue background indicates synteny between the Tr01 hapA chromosome and chromosomes in other haploid genomes, while the - symbol on a yellow background indicates the absence of synteny between the Tr01 hapA chromosome and chromosomes in other haploid genomes.

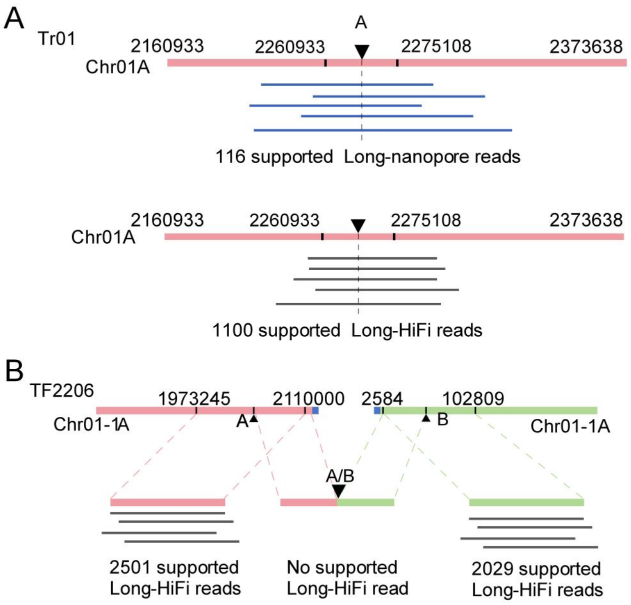

Supplementary Figure 8. Evidence for the validation of chromosomal rearrangement of Chr01. (A) Long-nanopore and Long-HiFi reads mapping on region flanked by breakpoint A of Tr01A. (B) Long-HiFi reads mapping on regions flanked by breakpoint A and B of TF2206A.

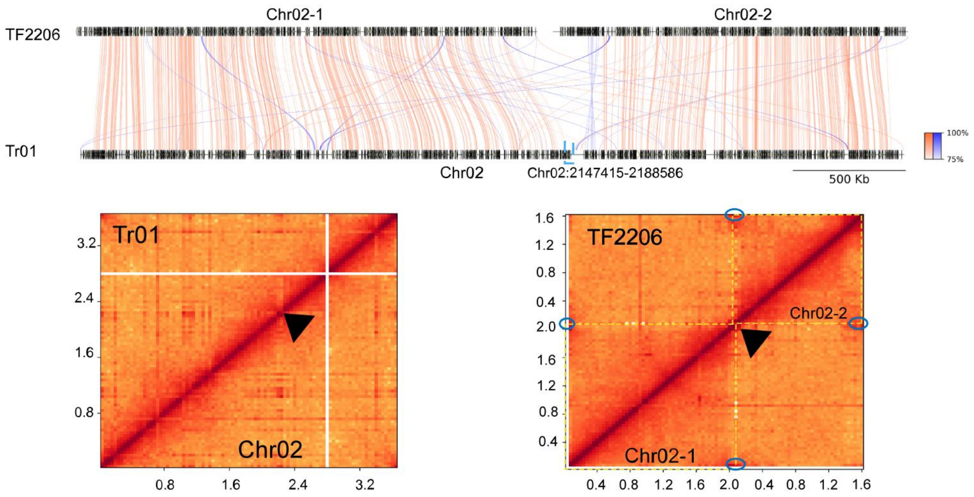

Supplementary Figure 9. Syntenic conservation analysis of Chr02 between Tr01 and TF2206 strains. Blue box delineates syntenic region boundaries.

Hi-C contact heatmaps of Chr02 (40-Kb resolution) are shown for Tr01 (left) and TF2206 (right). Blue triangles indicate syntenic boundaries, while solid black circles mark telomere interaction hotspots.

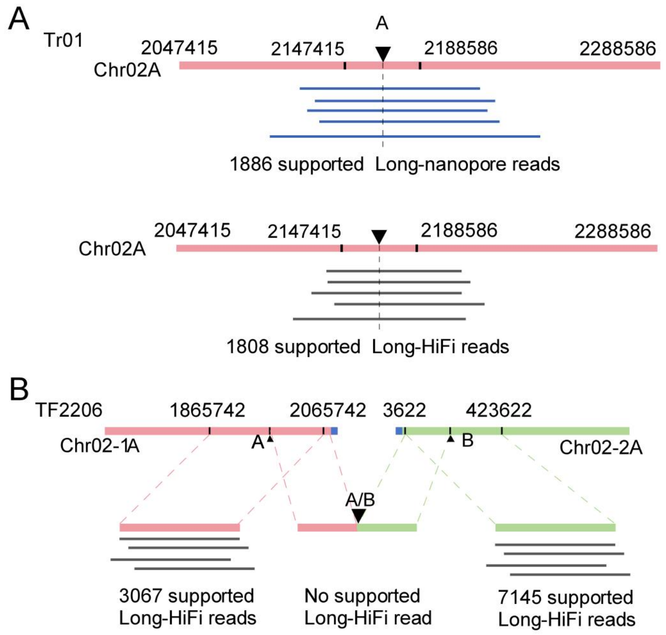

Supplementary Figure 10. Evidence for the validation of chromosomal rearrangement of Chr02.

(A) Long-nanopore and Long-HiFi reads mapping on region flanked by breakpoint A of Tr01A. (B) Long-HiFi reads mapping on regions flanked by breakpoint A and B of TF2206A.

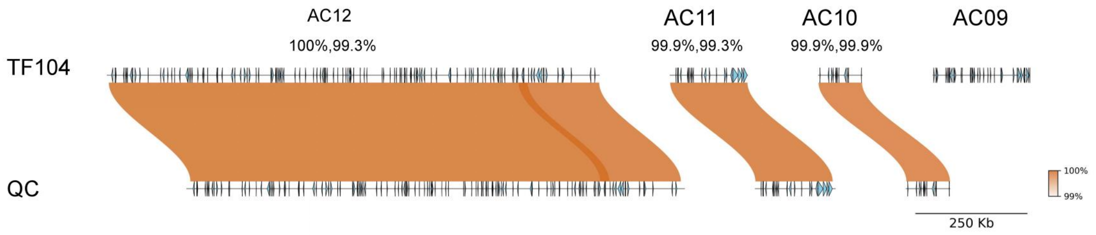

Supplementary Figure 11. Synteny analysis of accessory chromosomes in the QCA and TF104A genomes.

The homologous chromosomes AC10 to AC12 show near-identical synteny between the QCA and TF104A genomes, with sequence similarity >99.9% and coverage ≥99.3%.

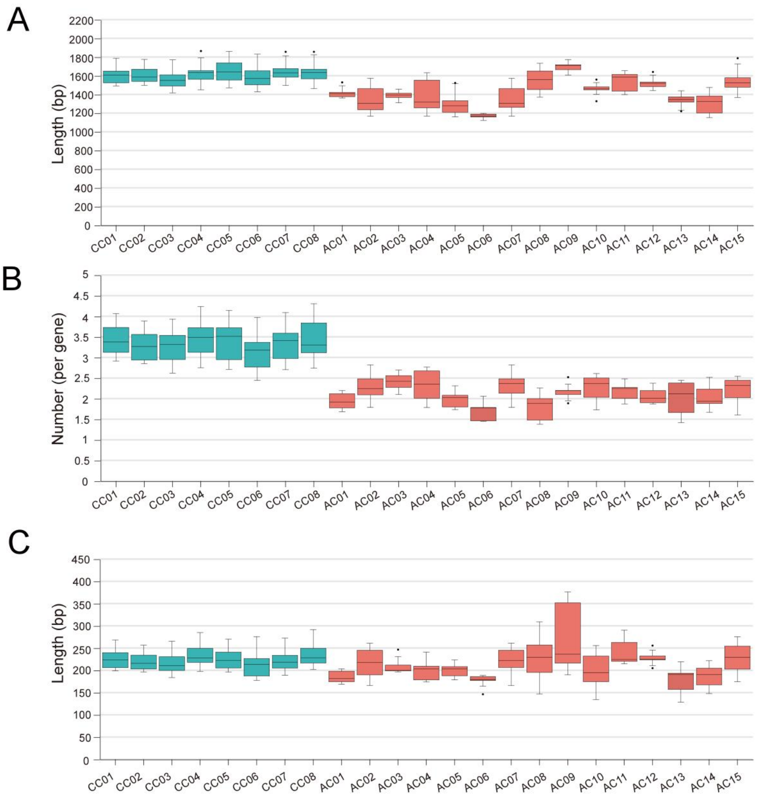

Supplementary Figure 12. Boxplot of gene length (A), number of introns per gene (B), and single intron length (C) in core chromosomes (CC01-CC08) and accessory chromosomes (AC01-AC15).

The sample size (n) indicates the number of chromosomes included in each group. Individual chromosomes analyzed within predefined chromosome groups represent the unit of study. Accordingly, the boxplot results primarily reflect overall distributional differences in the analyzed features among chromosome groups, rather than assuming complete statistical independence among individual chromosome data points. In each box plot, the central line represents the median, the box denotes the interquartile range (25th-75th percentiles), whiskers indicate the full data range (minimum to maximum values), and outliers are shown as individual points. All data points represent biological replicates; no technical replicates were used.Statistical definitions, sample sizes, and units of study are provided in Supplementary Table 4.

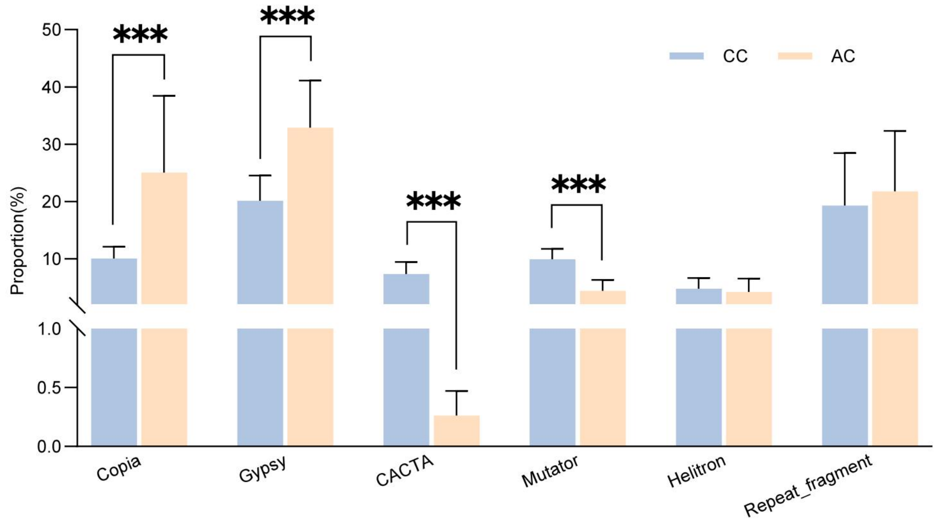

Supplementary Figure 13. Proportional comparison of six transposable element types between core chromosomes (CC) and accessory chromosomes (AC) in *T. fuciformis*.

Bars represent mean values ± SEM. The sample size (n) corresponds to the number of chromosomes included in each group (CC01-CC08, n = 8; AC01-AC15, n = 15). Statistical significance between CC and AC was assessed using a two-sided Student's t-test. ***$P < 0.001$; $P < 0.01$. Exact P-values are provided in the Source Data file.

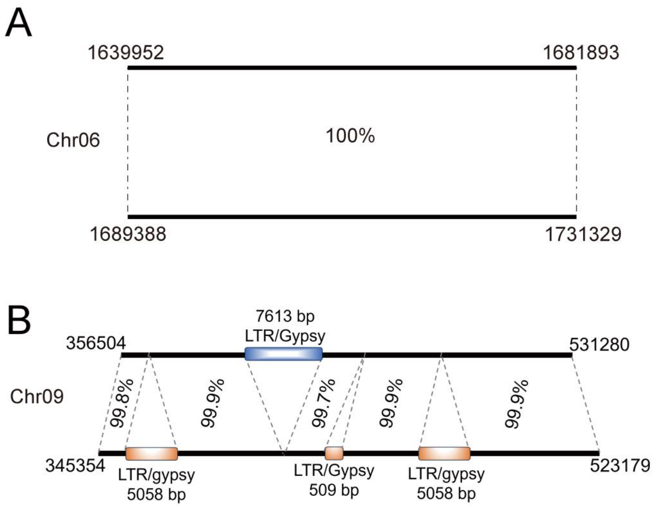

Supplementary Figure 14. Synteny analysis of Starship pairs: Chr06 (core, A) & Chr09 (accessory, B)
