# Supplementary material for: Polymorphism and evolutionary origins of accessory chromosomes in the basidiomycete Tremella fuciformis
Source: Nat Commun. 2026 Feb 27;17:3275. doi: 10.1038/s41467-026-70078-y (PMC13066641; doi:10.1038/s41467-026-70078-y)
Supplement: Supplementary file 2 — Reporting Summary [file 41467_2026_70078_MOESM2_ESM.pdf]

nature portfolio

Corresponding author(s): Baogui Xie Ray Ming Youjin Deng

Last updated by author(s): Jan 27, 2026

Reporting Summary

Nature Portfolio wishes to improve the reproducibility of the work that we publish. This form provides structure for consistency and transparency in reporting. For further information on Nature Portfolio policies, see our Editorial Policies and the Editorial Policy Checklist.

Statistics

For all statistical analyses, confirm that the following items are present in the figure legend, table legend, main text, or Methods section.

n/a	Confirmed	
☐	☒	The exact sample size (*n*) for each experimental group/condition, given as a discrete number and unit of measurement
☐	☒	A statement on whether measurements were taken from distinct samples or whether the same sample was measured repeatedly
☐	☒	The statistical test(s) used AND whether they are one- or two-sided<br>*Only common tests should be described solely by name; describe more complex techniques in the Methods section.*
☒	☐	A description of all covariates tested
☒	☐	A description of any assumptions or corrections, such as tests of normality and adjustment for multiple comparisons
☐	☒	A full description of the statistical parameters including central tendency (e.g. means) or other basic estimates (e.g. regression coefficient) AND variation (e.g. standard deviation) or associated estimates of uncertainty (e.g. confidence intervals)
☐	☒	For null hypothesis testing, the test statistic (e.g. *F*, *t*, *r*) with confidence intervals, effect sizes, degrees of freedom and *P* value noted<br>*Give P values as exact values whenever suitable.*
☒	☐	For Bayesian analysis, information on the choice of priors and Markov chain Monte Carlo settings
☒	☐	For hierarchical and complex designs, identification of the appropriate level for tests and full reporting of outcomes
☒	☐	Estimates of effect sizes (e.g. Cohen's *d*, Pearson's *r*), indicating how they were calculated

Our web collection on statistics for biologists contains articles on many of the points above.

Software and code

Policy information about availability of computer code

Data collection	For Tremella fuciformis, sequencing data collection was conducted as follows: (1) Second-generation sequencing data: Acquired via the Illumina NovaSeq 6000 platform (2×150 bp paired-end sequencing); (2) Third-generation sequencing and Hi-C data: Generated using PacBio CCS sequencing with the Sequel IIe platform.
Data analysis	HiFlasm(v0.25.3-r339),HiCPro (v2.10.0), HiCPlotter (v0.7.3), FUNANNOTATE (v1.8.16), DIAMOND (v2.1.8), InterProScan (v5.67-99.0), Pfam 36.0, SMART 9.0, MoBIDB Lite 2.0, Deep-MM/MM (v1.0), signalP 6.0, LTRharvest(GenomeTools V1.6.5),EDTA(V2.2.2),RepeatMasker (v4.0), MAFFT(V7.535),fastTree (v2.1.11), MEGA 11 (v11.0.13), DIAMOND (v2.1.8),OrthoFinder (v2.5.5), BWA-MEM (v0.7.18), GATK (v4.0.11.0), Samtools (v1.15.1), MUMmer (v4.0.0), PyGenomeViz (v1.5.0), OrthoANIu(https://www.ezbiocloud.net/tools/ani), MCScanX (https://github.com/wyp1125/MCScanX), UNITE database (https://unite.ut.ee/schedule_analysis.php), Starfish (v1.0.0), Chipplot(v2.6.1,https://www.chiplot.online/), excel2021, Python 3.8.10, GraphPad Prism 9.5.1, R 3.6.3, IBS (v1.0), PPT

For manuscripts utilizing custom algorithms or software that are central to the research but not yet described in published literature, software must be made available to editors and reviewers. We strongly encourage code deposition in a community repository (e.g. GitHub). See the Nature Portfolio guidelines for submitting code & software for further information.

Data

Policy information about availability of data

All manuscripts must include a data availability statement. This statement should provide the following information, where applicable:

- Accession codes, unique identifiers, or web links for publicly available datasets
- A description of any restrictions on data availability
- For clinical datasets or third party data, please ensure that the statement adheres to our policy

The sequencing reads of 15 Tremella fuciformis strains generated in this study have been deposited in NCBI BioProject PRJNA1247727 [https://www.ncbi.nlm.nih.gov/bioproject/PRJNA1247727].
Genomic sequences (mitochondrial and nuclear genomes) of these strains have been deposited at the National Genomics Data Center (NGDC) under accession PRJCA052213 [https://ngdc.cncb.ac.cn/search/all?q=PRJCA052213]. The mitochondrial genome sequence of Tremella fuciformis TF01 used in this study is available in the GenBank database under accession code NC_036422.1 [https://www.ncbi.nlm.nih.gov/nuccore/NC_036422.1]. The ITS sequence of Tremella fuciformis CBS 6970 used in this study is available in the GenBank database under accession code NR_155936.1 [https://www.ncbi.nlm.nih.gov/nuccore/NR_155936.1]. Source data are provided with this paper.

Research involving human participants, their data, or biological material

Policy information about studies with human participants or human data. See also policy information about sex, gender (identity/presentation), and sexual orientation and race, ethnicity and racism.

Reporting on sex and gender	Not applicable
Reporting on race, ethnicity, or other socially relevant groupings	Not applicable
Population characteristics	Not applicable
Recruitment	Not applicable
Ethics oversight	Not applicable

Note that full information on the approval of the study protocol must also be provided in the manuscript.

Field-specific reporting

Please select the one below that is the best fit for your research. If you are not sure, read the appropriate sections before making your selection.

[x] Life sciences [ ] Behavioural & social sciences [ ] Ecological, evolutionary & environmental sciences

For a reference copy of the document with all sections, see nature.com/documents/nr-reporting-summary-flat.pdf

Life sciences study design

All studies must disclose on these points even when the disclosure is negative.

Sample size	The sample size (16 strains of Tremella fuciformis) was selected based on the availability of field-collected specimens across regions/seasons, which sufficiently captures the genomic diversity in the studied areas.
Data exclusions	No data were excluded from the analyses
Replication	Reproducibility was verified via standard protocols for HiFi/Hi-C sequencing & assembly, and established bioinformatics pipelines for genomic analyses (assembly, scaffolding, annotation). Consistent results were obtained across analytical workflows.
Randomization	This study focuses on field-collected genomic specimens (no experimental group allocation), so randomization is not relevant.
Blinding	This study involves genomic sequencing & computational analysis (no experimental groups or human/animal subjects), so blinding is not relevant.

Reporting for specific materials, systems and methods

We require information from authors about some types of materials, experimental systems and methods used in many studies. Here, indicate whether each material, system or method listed is relevant to your study. If you are not sure if a list item applies to your research, read the appropriate section before selecting a response.

Materials & experimental systems

n/a	Involved in the study	
☒	☐	Antibodies
☒	☐	Eukaryotic cell lines
☒	☐	Palaeontology and archaeology
☐	☒	Animals and other organisms
☒	☐	Clinical data
☒	☐	Dual use research of concern
☒	☐	Plants

Methods

n/a	Involved in the study	
☒	☐	ChIP-seq
☒	☐	Flow cytometry
☒	☐	MRI-based neuroimaging

Animals and other research organisms

Policy information about studies involving animals; ARRIVE guidelines recommended for reporting animal research, and Sex and Gender in Research

Laboratory animals	The study did not involve laboratory animals
Wild animals	The study did not involve Wild animals
Reporting on sex	Sex is not relevant to this study as the research organism is Tremella fuciformis (a fungus), and sex-based analysis was not performed.
Field-collected samples	Field-collected samples for this study consist of 16 strains of Tremella fuciformis (a basidiomycete fungus) collected from different regions across spring, summer, and autumn. Following collection, samples were maintained under controlled laboratory conditions (25°C, dark) on potato dextrose agar (PDA) medium. At the conclusion of the experiment, samples were preserved for subsequent analyses.
Ethics oversight	No ethical approval was required as the study involves a fungus (Tremella fuciformis) and does not involve animals or human subjects.

Note that full information on the approval of the study protocol must also be provided in the manuscript.

Plants

Seed stocks	This study does not involve plants, so no seed stocks or plant materials are included. The research focuses on Tremella fuciformis (a fungus), with field-collected samples as described in prior sections.
Novel plant genotypes	This study does not involve plants, so no novel plant genotypes were generated. The research organism is Tremella fuciformis (a fungus), and plant genotype generation methods are inapplicable here.
Authentication	This study does not involve plants, so no authentication procedures for seed stocks or novel plant genotypes were performed. The research focuses on Tremella fuciformis (a fungus), and plant authentication methods are irrelevant to this work
